# Evaluating the internal architecture of the postnatal human maxilla across the stages of tooth development and eruption

**DOI:** 10.1007/s00276-026-03980-1

**Published:** 2026-08-28

**Authors:** Xolisiwe Mhlathi, Candice Small, Erin F. Hutchinson

**Affiliations:** Human Identification and Variation Research Unit, Department of Anatomical Sciences, School of Biomedical Sciences, Faculty of Health Sciences, University of the Witwatersrand, Johannesburg, South Africa

**Keywords:** Micro-CT, Trabecular bone, Dentition, Growth, Maxilla

## Abstract

**Purpose:**

General growth as well as functional demands that are associated with developmental needs of young individuals are said to contribute to the changes in micro-architecture of maxilla. However, little is known about the nature of these changes on the maxilla, particularly across the different stages of dental development and eruption. Thus, this study aimed to assess variations in micro-architecture of the alveolar region of the human sub-adult and adult maxilla across different stages of dental development and eruption.

**Methods:**

The study sample included seventy-nine individuals, subdivided into three dentition groups: deciduous dentition (*n* = 28), mixed dentition (*n* = 9), and permanent dentition (*n* = 42). Maxillae were scanned using micro-computed tomography. Seven regions were selected in each dental crypt for evaluation of micro-architecture which included bone volume ratio, bone surface to bone volume ratio, trabecular thickness, trabecular number, and trabecular spacing.

**Results:**

The micro-architecture of the maxilla changed from a more porous to a less porous nature, following patterns of remodeling that are due to the general process of bone growth and related functional demands. These changes included an increase in bone volume fraction while bone surface to volume ratio and trabecular number both significantly decreased.

**Conclusion:**

The observed changes reflect the normal growth differences, the state of the dentition, as well as the functional adaptation of the oral cavity.

## Introduction

 The remodeling of both the human maxilla and the mandible are shaped by general skeletal growth and functional demands associated with development [3]. These functional demands result from tooth development and eruption, increased forces on muscle attachment sites and changes in the functional environment with growth such as the transition from suckling to mastication [3, 12, 27]. These factors that are as a result of the general growth of the maxilla are thought to influence the underlying bone micro-architecture of the maxilla by thickening the alveolar ridge and changing the trabecular morphology to a more compact structure [7, 27, 30, 31, 40, 62]. However, there is a paucity of knowledge addressing how growth as well as its associated demands may manifest in the underlying bone across the different states of the dentition. Knowledge of the changes in the underlying micro-architecture of maxilla across the stages of the dentition will aid in understanding the anatomy of the juvenile maxilla and how it changes during growth.

The maxilla forms by the process of intramembranous ossification, where mesenchymal tissue is directly converted to bone [37, 58]. The ossification centers of the maxilla are observed around the sixth week of intrauterine life and are located above the dental lamina that gives rise to the developing dentition [15, 58]. In the eighth week of intrauterine life, the body of the maxilla and its four processes, which include the frontal, zygomatic, palatine, and alveolar processes, are identifiable. The maxillary dentition develops mesiodistally in the alveolar process and starts developing at approximately six weeks of intrauterine life and continues until early adulthood (~18 years) [15, 24, 57, 58, 61]. The deciduous dentition starts erupting at approximately six months postnatally and continues until approximately three years postnatally, when all the deciduous teeth have erupted apart from the third molars, which are thought to erupt between six and 12 years [4, 19, 41, 58]. This period of growth is characterized by shedding of deciduous teeth and replacement by their permanent successors [4, 19]. The last permanent tooth, the third molar, does not fully erupt until early adult life and displays a great amount of variation between populations [58]. In line with staged development and eruption of the dentition, it is important to gain knowledge into the interplay between how the supporting bony architecture changes relative to the individual teeth as well as across various states of the dentition.

The prenatal growth of the maxilla is influenced by the growth of associated structures, such as the appearance of the incisive fissure which plays a role in accommodating the increasing volume of tooth germs in the alveolar process [58]. In addition, the development of the maxillary sinus plays a major role in increasing the height of the maxilla [15]. Postnatally, the maxilla grows rapidly, and its growth is related to the functional areas of the viscerocranium, including the orbit and its contents as well as the nasal cavity and associated paranasal sinuses [18, 58]. The major influence of maxillary growth and changes in internal micro-architecture postnatally is the requirement for space to accommodate the increasing size and number of the teeth, which are associated with changes in functional environment such as the shift from suckling to mastication [12, 58]. Demonstrating the importance of the relationship between the maxillary alveolar bone and the dentition, the proper alveolar ridge is only observed in the presence of teeth and is resorbed and greatly reduced when teeth are lost [22, 28, 58, 65].

The changes in micro-architecture of the maxilla are not only related to the development and eruption of dentition, but also to the functional demands of the oral cavity [3, 64]. The adaptation of the maxilla to these functional demands is not well understood due to its structural complexity [50]. The complexity of the maxilla results from the features associated with it including the sutures and the paranasal sinuses [50, 58]. Maxillary morphology allows for several functions that are associated with development, including swallowing, the senses of smell and sight, respiration, and anchoring muscles of facial expression and a part of the masseter muscle [18, 50, 58]. Occlusal forces resulting from mastication are exerted on the teeth and are transferred to the alveolar bone by the associated periodontal ligament [17, 47, 71]. These forces are then converted into compression vectors around the root surfaces of the teeth and result in the remodeling of the alveolar bone in this region. The result of this remodeling is the trabeculae radiating from the root and this arrangement helps limit the shear stress on the alveolar ridge [71]. The facial bones have a macro-structure that is suited to enable maximal strength with minimal mass [70, 71]. In accordance with this principle, the loads on maxillary teeth will not only affect the micro-architecture of the alveolar bone, but also other associated areas including the orbits, and paranasal sinuses and the attachment sites for the muscles of mastication [70]. Biomechanical stresses result in trabecular bone remodeling, changing its micro-architecture [34, 71].

While valuable information on the trabecular micro-architecture of the maxilla is provided in the literature, studies are limited to older age groups [21, 28, 45, 65]. This limits the depth of inferences which can be made regarding changes in the trabecular micro-architecture that take place during important stages of juvenile and sub-adult growth. These stages include changes in the functionality of structures associated with the maxilla i.e., dental development and eruption as well as general growth of the maxilla. Therefore, this study aimed to assess the changes in micro-architecture of the alveolar region of the postnatal and subadult human maxilla in relation to dental development and eruption. This study hypothesizes that the micro-architecture of the maxilla will change across dentition groups from a more porous to a less porous nature, following modeling and remodeling patterns due to normal growth patterns and functional demand responses.

## Materials and methods

### Sample description

Maxillae from 79 individuals were sourced from the Raymond A. Dart Collection of Modern Human Skeletons housed at the Department of Anatomical Sciences, School of Biomedical Sciences, University of the Witwatersrand, Johannesburg, South Africa. The individuals used for this study were aged between birth and 18 years and were assigned to three dentition groups based on dental development and eruption standards proposed by the London Atlas of Human Tooth Development and Eruption [4] and the WITS Atlas of Tooth Formation and Emergence [19]. The three sample groups included individuals with deciduous dentition only (*n* = 28; F = 14 and M = 14; 0–5y), mixed dentition (*n* = 9; F = 4 and M = 5; 6–12y), and permanent dentition only (*n* = 42; F = 10 and M = 32; 13–18y). Due to the small sample number in the mixed dentition group, the different potential combinations existing within the mixed dentition were not explored, i.e., the absence of anterior teeth versus posterior teeth. Individuals were excluded if the maxillae were broken on both the left and the right side or showed any signs of obvious trauma, surgical repair, and orthodontic treatments. The study maxillae were used in accordance with the National Health Act 61 of 2003.

### Ethics

Permission to use an ethics clearance waiver previously issued to the Department of Anatomical Sciences, University of the Witwatersrand was obtained (Certificate number: W-CBP-220504-01). The ethical clearance certificate is available on request.

### Scanning and reconstruction

The maxillae were scanned at the MIXRAD laboratory of the South African Nuclear Energy Corporation (NECSA) using the Nikon XTH 225 L micro-focus CT X-ray unit (Nikon Metrology, Belgium). Each cranium was securely placed in a polystyrene mold, which prevented the movement of the skull during scanning. Subsequent to scanning preparations, the mounted skulls were placed onto a rotating sample manipulator to allow for scanning in 360 degrees. The following scanning parameters were put in place: 100 kV/100 µA, at 0.5 frame per second exposure, 1000 projections and 125 μm resolution. The scans were reconstructed using Nikon CTPro software version XT4.4.3 (Nikon Metrology, Belgium).

### Analysis of scans

Scans were analyzed using VGStudio V3.2.3 (Volume Graphics GmbH, Germany). The reconstructed scans were first subjected to a surface determination analysis using an automatic setting for surface determination on VGStudio to enable clear determination of the surface landmarks of each skull and further filtering steps were not necessary for this sample. To facilitate the analysis of the dental crypt sides, the orientation of the maxillary slices were standardized by aligning every slice with an established plane. A transverse reference plane was used for all the scans to align slices to an anatomical plane by selecting four fiducial landmarks along the skull. The fiducial points selected were the right porion, the right nariale, the left nariale, and the left porion.

All the dental crypts on the left side of the maxilla for each individual were included in this study, except for the third molar crypt. The exclusion of the third molar was because it is the most variable amongst individuals in terms of position and timing development and eruption [20, 38, 55, 59, 60]. In cases where the dental crypts on the left side were damaged, the corresponding crypts on the right side were used for analysis. In cases where the teeth were missing on the left side of the maxilla, analysis was still conducted, provided that the entire crypt was intact. When considering the mixed dentition, in cases where there was a deciduous and a permanent dental crypt, the overall outside border of the two crypts combined was considered.

**Fig. 1:**
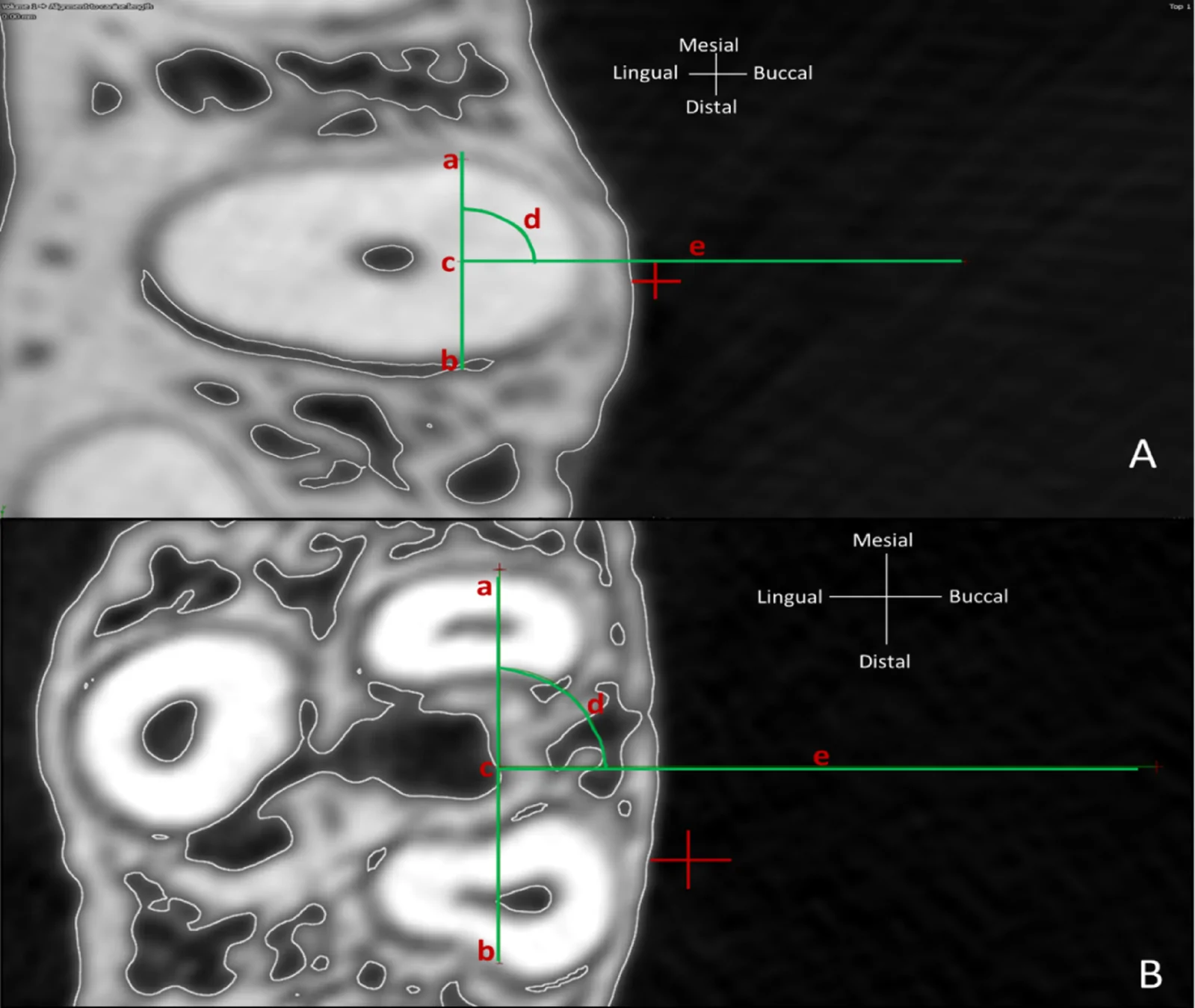
Micro-CT images of maxillary permanent central incisor (**A**) and deciduous first molar (**B**) crypts from the inferior view. Line ab is the maximum width of the crypt, point c is the mid-point of the crypt, and the buccolingual plane is marked by line e

To ensure repeatability of obtaining the midpoint sagittal section of each dental crypt, the maximum width (Fig. 1, line ab) was first taken at a level where bone was evident across at least three sides of the crypt i.e., between the mesial and the distal and parallel to the buccal side of the dental crypt opening of the maxilla across a single cross section. The midpoint (line ac) of the maximum width (line ab) was taken (Fig. 1). A 90-degree angle (angle d) was then put in place at the midpoint of the crypt (perpendicular to the maximum width, line ab). This was followed by a buccolingual plane through the crypt aligned with the 90-degree angle, indicated by line e (Fig. 1). Slices were then aligned with line e to give the sagittal section of each dental crypt. The same measurements in every crypt were taken to find the sagittal plane of the crypts for the entire study sample.

To analyze the trabecular micro-architecture of the left maxillary dental crypts across the selected sagittal section, seven volume of interests (VOIs) across the buccal and lingual sides of each dental crypt, as well as at the apex, were taken. The seven VOIs were done to ensure that all crypt sides were represented. The VOI of each dental crypt included inferior, middle, and superior regions across the buccal and lingual side respectively, and the apex regions [27]. The inferior volume of interest of each side was taken at the alveolar bony crest corresponding to the opening of each dental crypt (buccal inferior, BI; lingual inferior, LI). In the case of individuals with unerupted teeth, the inferior volume of interest was aligned with the crown of the erupting tooth on both the buccal and the lingual sides. The superior volume of interest of the buccal and the lingual sides were aligned with the apical side of the dental crypt (buccal superior: BS; lingual superior: LS). The middle volume of interest was taken halfway between the inferior and the superior volumes of interest (buccal middle: BM; lingual middle: LM). The apical volume of interest was taken at the deepest point on the apex of each dental crypt (apex region: AR). In cases of multirooted teeth where two roots were observed in the sagittal plane, two apical volumes of interest (buccal apex region: BA; lingual apex region: LA) were taken at the deepest point of each root. The values of all the trabecular micro-architecture parameters of the buccal and lingual apex volumes of interest were then averaged to get single apex values for every parameter of every multirooted crypt for comparison across all dental crypts. The volumes of interest were selected using a 2D disk selection tool. The diameter of the disc as well as the depth were set at 1 mm.

The internal micro-architecture of the bone at each volume of interest of every dental crypt was investigated using trabecular number (Tb.N) (1/mm), trabecular thickness (Tb.Th) (mm), trabecular spacing (Tb.Sp) (mm), bone surface to bone volume ratio (BS/BV) (mm^−1), and bone volume fraction (BV/TV) [3]. Tb.N refers to the number of trabeculae found within a selected region of the bone. Tb.Th is the average thickness of trabeculae in a selected region on the bone. Tb.Sp is the average distance between trabeculae [7, 49, 62]. BS/BV is the amount of surface area per unit volume of bone sample [33]. BV/TV measures the amount of mineralized bone per unit volume of the total bone sample [36]. The internal micro-architecture values were obtained using parameter calculation algorithm tool for morphometrics in VGStudio Max V3.2.3 (Volume Graphics GmbH, Germany).

### Data entry, management, and repeatability

The data collected was entered into Microsoft Excel (Microsoft Corporation, USA) and analyzed using IBM SPSS Statistics v.28.0 (IBM, USA) and R Studio v.4.2.2 (Posit Software, PBC, USA). Levels of repeatability were evaluated using two methods for comparison purposes: Lin’s Concordance Correlation Coefficient (Lin’s CCC) of Reproducibility [35] and Technical Error of Measurement (TEM) [25]. For intra-observer repeatability, trabecular parameters from nine individuals (~10% of the total sample) were selected from all the dentition groups across the sample and were analyzed by the primary observer and were repeated a week later. For inter- observer error repeatability, both the primary and the secondary observers analyzed the same trabecular parameters from the same 9 individuals. Repeatability was measured by calculating the degree of agreement between intra-observer and interobserver trabecular data using Lin’s CCC and TEM. Repeatability values of more than 0.90 were considered excellent.

### Statistical analysis

A Shapiro-Wilk’s test was used to assess the distribution of the data. Kruskal-Wallis and Dunn's post-hoc analyses were done to compare statistical differences in trabecular parameters between the three dentition groups across apical, buccal, and lingual sides of dental crypts. Descriptive statistics were calculated as median (interquartile range (IQR)) because the data was found to be non-normally distributed.

## Results

High levels of repeatability were observed for intra-observer error (82.9–97.1%) and inter-observer error (73.9–96.4%) for all the trabecular parameters except for trabecular thickness where the moderate repeatability was 46.3% for inter-observer and 49.9% for intra-observer. Using TEM, moderate to high levels of repeatability were observed for intra-observer (66.9–93.1%) and inter-observer (71.4–87.4%) and the lowest values for both intra- and inter-observer error were observed in trabecular thickness (67.0% and 71.0%, respectively).

## Patterns and distribution of micro-architecture between dentition groups

**Fig. 2:**
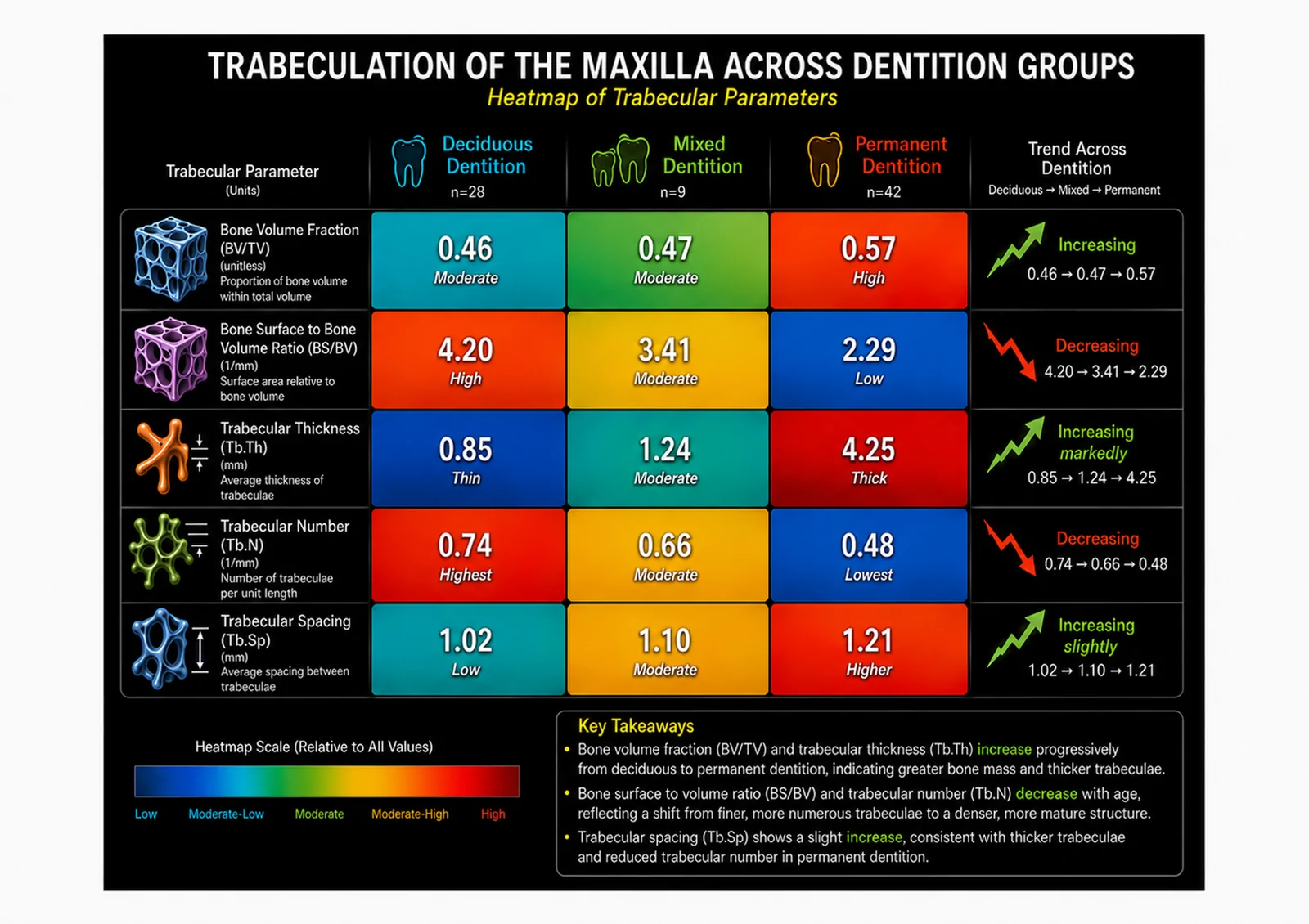
The mean patterns of trabecular micro-architecture parameters between the deciduous dentition group, mixed dentition group, and permanent dentition group. BV/TV, bone volume fraction; BS/BV, bone surface to bone volume ratio; Tb.Th, trabecular thickness; Tb.N, trabecular number; Tb.Sp, trabecular spacing

Mean trabecular parameters varied across groups when observed visually (Fig. 2). Bone volume fraction and trabecular spacing increased slightly from deciduous to permanent dentition group, while trabecular thickness increased considerably (Fig. 2). Bone surface to bone volume ratio and trabecular number both decreased from deciduous to permanent dentition groups (Fig. 2). The distribution of the trabecular parameters within and between dentition groups is presented as median (IQR) in Tables 1, 2, 3, 4 and 5.

**Fig. 3:**
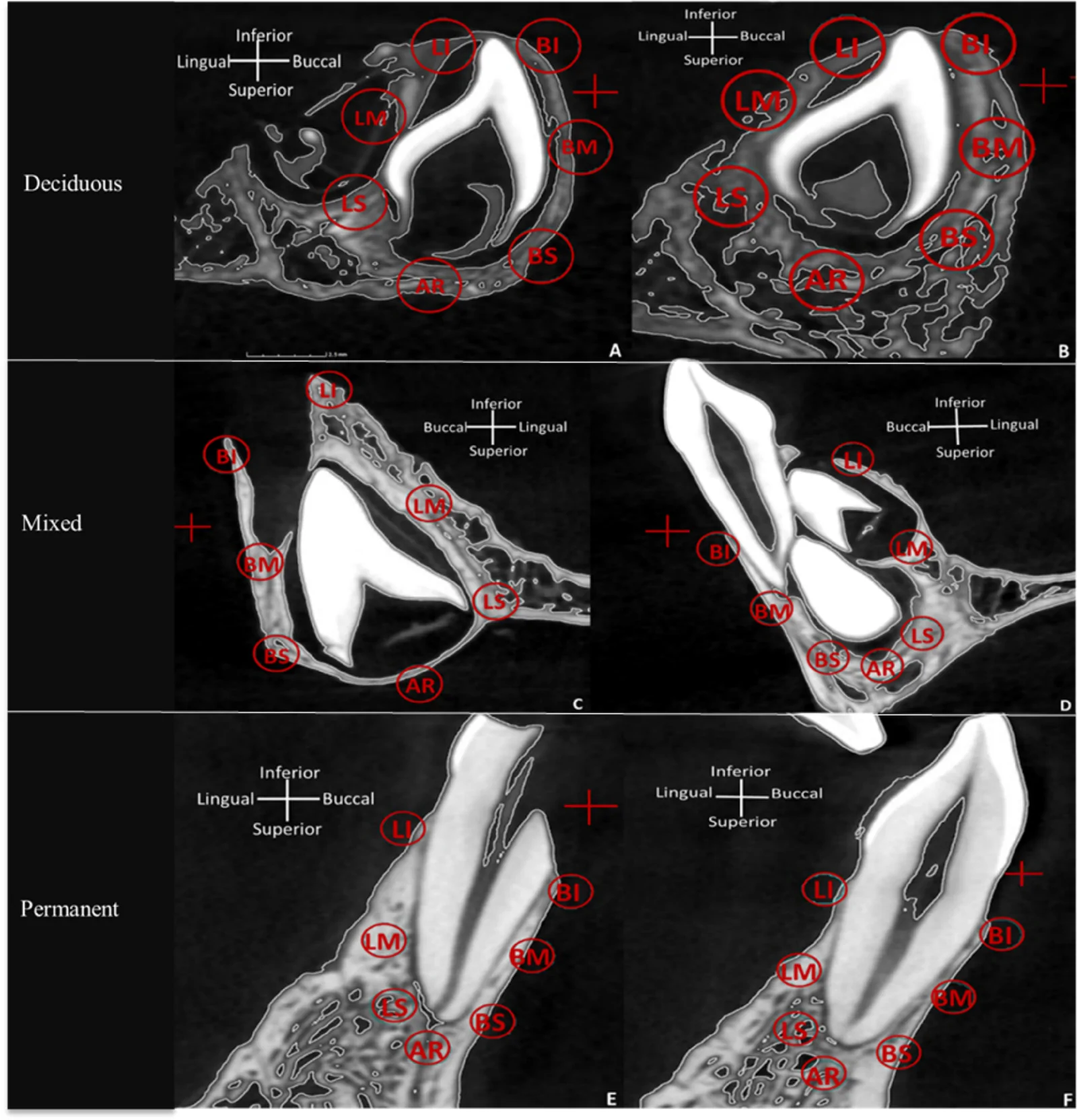
Micro-CT images showing central incisors (**A**,** C**,** E**) and lateral incisors (**B**,** D**,** F**) from the deciduous, mixed, and permanent dentition groups, respectively.aApex region, AR; lingual superior, LS; lingual middle, LM; lingual inferior, LI; buccal superior, BS; buccal middle, BM, and buccal inferior, BI)

**Table 1:** Descriptive statistics of bone volume fraction (BV/TV) for each dental crypt side across dentition groups

	Median (IQR)										
	CI	LI	C	DM1	DM2	PM1	PM2			M1	M2
Deciduous (*n* = 28; 0–5 years)											
AA	0.58 (0.37)	0.64 (0.23)	0.52 (0.31)	0.51 (0.24)	0.41 (0.32)	−	−			0.21 (0.32)	−
BA	0.23 (0.16)	0.50 (0.31)	0.37 (0.24)	0.38 (0.11)	0.32 (0.23)	−	−			0.37 (0.24)	−
LA	0.58 (0.24)	0.51 (0.26)	0.52 (0.19)	0.48 (0.22)	0.47 (0.33)	−	−			0.47 (0.40)	−
Mixed (*n* = 9; 6–12 years)											
AA	0.52 (0.35)	0.49 (0.51)	0.65 (0.23)	0.54 (0.24)	0.35 (0.28)	−	−			0.36 (0.31)	−
BA	0.44 (0.19)	0.43 (0.29)	0.44 (0.12)	0.40 (0.17)	0.52 (0.24)	−	−			0.43 (0.20)	−
LA	0.55 (0.19)	0.49 (0.25)	0.52 (0.27)	0.45 (0.27)	0.47 (0.24)	−	−			0.45 (0.15)	−
Permanent (*n* = 42; 13–18 years)											
AA	0.91 (0.20)	0.80 (0.30)	0.84 (0.40)	−	−	0.70 (0.31)	0.62 (0.35)			0.63 (0.34)	0.60 (0.26)
BA	0.36 (0.25)	0.42 (0.24)	0.34 (0.23)	−	−	0.36 (0.22)	0.54 (0.27)			0.66 (0.25)	0.65 (0.29)
LA	0.65 (0.15)	0.57 (0.19)	0.60 (0.19)	−	−	0.58 (0.22)	0.57 (0.18)			0.62 (0.19)	0.59 (0.21)

IQR, interquartile range; CI, central incisor; LI, lateral incisor; C, canine; DM1, deciduous molar 1; DM2, deciduous molar 2; PM1, premolar 1; PM2, premolar 2; M1, molar 1; M2, molar 2; AA, apical average; BA, buccal average; LA, lingual average

**Table 2:** Descriptive statistics of bone surface to bone volume (BS/BV) for each dental crypt side across dentition groups

Median (IQR)									
	CI	LI	C	DM1	DM2	PM1	PM2	M1	M2
Deciduous (*n* = 28; 0–5 years)									
AA	3.89 (2.35)	3.32 (2.58)	3.86 (2.35)	4.46 (2.94)	5.44 (3.60)	−	−	5.26 (6.15)	−
BA	4.49 (1.99)	3.07 (2.17)	4.20 (2.89)	3.85 (2.15)	5.96 (3.57)	−	−	5.52 (4.51)	−
LA	2.89 (2.02)	3.32 (2.25)	3.86 (2.29)	3.79 (2.97)	3.94 (3.04)	−	−	4.12 (4.59)	−
Mixed (*n* = 9; 6–12 years)									
AA	3.54 (2.96)	4.28 (6.05)	2.66 (1.42)	3.86 (1.60)	5.14 (4.66)	−	−	3.80 (3.30)	−
BA	3.20 (1.88)	3.16 (2.07)	2.51 (1.11)	2.76 (1.80)	3.54 (4.59)	−	−	3.58 (2.47)	−
LA	3.56 (2.36)	3.09 (1.87)	2.74 (1.99)	2.92 (1.54)	3.07 (1.03)	−	−	2.78 (0.85)	−
Permanent (*n* = 42; 13–18 years)									
AA	0.81 (1.64)	1.82 (2.96)	1.32 (2.68)	−	−	2.09 (1.79)	2.55 (2.35)	2.89 (2.54)	2.69 (2.07)
BA	2.71 (1.48)	2.21 (1.43)	2.60 (1.72)	−	−	2.16 (0.96)	1.61 (1.05)	1.92 (2.02)	1.75 (1.32)
LA	2.07 (1.52)	2.30 (1.58)	2.29 (2.02)	−	−	1.85 (1.41)	2.38 (1.58)	1.47 (1.17)	1.61 (1.05)

IQR, interquartile range; CI, central incisor; LI, lateral incisor; C, canine; DM1, deciduous molar 1; DM2, deciduous molar 2; PM1, premolar 1; PM2, premolar 2; M1, molar 1; M2, molar 2; AA, apical average; BA, buccal average; LA, lingual average

**Table 3:** Descriptive statistics of trabecular thickness (Tb.Th) for each dental crypt side across dentition groups

Median (IQR)										
	CI	LI	C	DM1	DM2	PM1	PM2		M1	M2
Deciduous (*n* = 28; 0–5 years)										
AA	0.52 (0.24)	0.61 (0.45)	0.51 (0.46)	0.45 (0.34)	0.37 (0.23)	–	–		0.35 (0.23)	–
BA	0.48 (0.25)	0.92 (0.91)	0.51 (0.43)	0.64 (0.32)	0.37 (0.25)	–	–		0.40 (0.26)	–
LA	0.91 (0.65)	0.68 (0.53)	0.61 (0.33)	0.64 (0.45)	0.58 (0.65)	–	–		0.59 (0.78)	–
Mixed (*n* = 9; 6–12 years)										
AA	0.50 (0.44)	0.46 (0.79)	0.76 (0.29)	0.58 (0.25)	0.38 (0.41)	–	–		0.62 (0.58)	–
BA	0.71 (0.60)	0.76 (0.68)	0.95 (0.49)	0.75 (0.60)	0.67 (0.58)	–	–		0.58 (0.38)	–
LA	0.67 (0.19)	0.90 (0.46)	0.77 (0.66)	0.83 (0.54)	0.78 (0.21)	–	–		0.80 (0.37)	–
Permanent (*n* = 42; 13–18 years)										
AA	1.92 (3.70)	1.06 (1.58)	0.94 (2.73)	–	–	1.00 (1.35)	0.88 (1.04)		0.75 (1.10)	0.80 (0.69)
BA	0.81 (0.58)	1.11 (0.70)	0.95 (0.68)	–	–	1.06 (0.55)	1.47 (1.00)		1.30 (1.50)	1.30 (1.02)
LA	1.26 (1.65)	1.34 (1.18)	1.17 (1.60)	–	–	1.52 (1.27)	1.05 (0.70)		1.84 (0.97)	1.57 (1.27)

IQR, interquartile range; CI, central incisor; LI, lateral incisor; C, canine; DM1, deciduous molar 1; DM2, deciduous molar 2; PM1, premolar 1; PM2, premolar 2; M1, molar 1; M2, molar 2; AA, apical average; BA, buccal average; LA, lingual average

**Table 4:** Descriptive statistics of trabecular number (Tb.N) for each dental crypt side across dentition groups

Median (IQR)										
	CI	LI	C	DM1	DM2	PM1	PM2		M1	M2
Deciduous (*n* = 28; 0–5 years)										
AA	0.59 (0.41)	1.05 (0.50)	0.95 (0.40)	1.04 (0.52)	1.00 (0.51)	–	–		0.99 (0.65)	–
BA	0.45 (0.20)	0.58 (0.37)	0.59 (0.29)	0.69 (0.46)	0.71 (0.61)	–	–		0.89 (0.26)	–
LA	0.75 (0.26)	0.68 (0.29)	0.90 (0.26)	0.77 (0.32)	0.76 (0.41)	–	–		0.75 (0.33)	–
Mixed (*n* = 9; 6–12 years)										
AA	0.83 (0.38)	0.97 (0.58)	0.96 (0.26)	0.89 (0.34)	0.77 (0.29)	–	–		0.69 (0.16)	–
BA	0.55 (0.29)	0.44 (0.40)	0.40 (0.27)	0.64 (0.31)	0.86 (0.16)	–	–		0.78 (0.21)	–
LA	0.83 (0.32)	0.66 (0.24)	0.67 (0.26)	0.54 (0.25)	0.58 (0.28)	–	–		0.69 (0.21)	–
Permanent (*n* = 42; 13–18 years)										
AA	0.36 (0.55)	0.70 (0.68)	0.41 (0.63)	–	–	0.62 (0.41)	0.69 (0.42)		0.82 (0.54)	0.77 (0.58)
BA	0.34 (0.16)	0.31 (0.13)	0.30 (0.14)	–	–	0.31 (0.07)	0.34 (0.14)		0.54 (0.43)	0.52 (0.31)
LA	0.55 (0.33)	0.52 (0.23)	0.56 (0.27)	–	–	0.46 (0.20)	0.54 (0.23)		0.32 (0.23)	0.36 (0.19)

IQR, interquartile range; CI, central incisor; LI, lateral incisor; C, canine; DM1, deciduous molar 1; DM2, deciduous molar 2; PM1, premolar 1; PM2, premolar 2; M1, molar 1; M2, molar 2; AA, apical average; BA, buccal average; LA, lingual average

**Table 5:** Descriptive statistics of trabecular spacing (Tb.Sp) for each dental crypt side across dentition groups

Median (IQR)																			
		CI		LI		C		DM1		DM2		PM1		PM2			M1		M2
Deciduous (*n* = 28; 0–5 years)																			
AA		0.84 (0.99)		0.31 (0.18)		0.45 (0.34)		0.40 (0.35)		0.54 (0.60)		–		–			0.80 (0.91)		–
BA		0.87 (1.40)		0.98 (1.29)		1.51 (0.89)		1.32 (1.01)		1.04 (1.29)		–		–			0.77 (0.62)		–
LA		0.61 (0.34)		0.82 (0.57)		0.59 (0.35)		0.82 (0.51)		0.81 (0.46)		–		–			0.65 (0.42)		–
Mixed (*n* = 9; 6–12 years)																			
AA		0.47 (0.38)		0.56 (0.72)		0.49 (0.32)		0.48 (0.28)		0.78 (0.82)		–		–			0.87 (0.69)		–
BA		1.16 (0.92)		1.57 (1.20)		1.32 (1.15)		1.20 (0.61)		0.76 (0.53)		–		–			0.97 (0.32)		–
LA		0.57 (0.43)		1.11 (0.75)		0.74 (0.46)		1.09 (1.23)		1.31 (1.03)		–		–			0.97 (0.55)		–
Permanent (*n* = 42; 13–18 years)																			
AA		0.25 (0.18)		0.29 (0.21)		0.31 (0.46)		–		–		0.43 (0.36)		0.43 (0.30)			0.42 (0.32)		0.47 (0.27)
BA		1.91 (1.23)		1.78 (1.18)		2.10 (1.11)		–		–		2.01 (1.06)		1.16 (1.03)			0.77 (0.54)		0.70 (0.71)
LA		0.79 (0.48)		0.95 (0.67)		0.88 (0.64)		–		–		0.94 (0.59)		0.87 (0.47)			1.05 (0.71)		1.03 (0.78)

IQR, interquartile range; CI, central incisor; LI, lateral incisor; C, canine; DM1, deciduous molar 1; DM2, deciduous molar 2; PM1, premolar 1; PM2, premolar 2; M1, molar 1; M2, molar 2; AA, apical average; BA, buccal average; LA, lingual average

### Bone micro-architecture variables between dentition groups

Significant results were observed for trabecular parameters between groups, except for trabecular spacing (Tb.Sp) on the buccal side, where no significant differences were observed (H = 3.53, *p* = 0.171, Table 6).

Bone volume fraction was significantly different across dentition groups in apical, buccal, and lingual sides (*p* ≤ 0.001, Table 6). Permanent dentition group consistently had a higher bone volume fraction when compared to the mixed dentition group across all crypt sides (*p* < 0.001, Table 7). Both deciduous and mixed dentition groups showed relatively similar mean ranks and no significant differences across the apical, buccal, and lingual sides (Tables 6 and 7).

There were significant differences in bone surface to bone volume ratio across all crypt sides between dentition groups (*p* ≤ 0.001, Table 6). Bone surface to bone volume ratio was significantly higher on the buccal side in the deciduous dentition group when compared to the buccal side of the mixed dentition group (*p* = 0.04, Table 7). No significant differences were observed on the apical and lingual sides between deciduous and mixed dentition groups. The permanent dentition group showed significantly lower bone surface to bone volume ratio values across all sides when compared to the mixed dentition group (*p* < 0.001, Table 7).

Trabecular thickness differed significantly across all sides between the dentition groups (*p* ≤ 0.001, Table 6). There were no significant differences in trabecular thickness across all sides between deciduous and mixed dentition groups (*p* > 0.05, Table 7). However, trabecular thickness was significantly higher in the permanent dentition groups across all sides when compared to the mixed dentition group (*p* < 0.001, Table 7).

Trabecular number differed significantly across all sides between dentition groups (*p* ≤ 0.001, Table 6). A significant difference was observed on the lingual side, where deciduous dentition group exhibited a higher trabecular number when compared to the mixed dentition group (*p* = 0.003, Table 7). There were no significant differences between deciduous and mixed dentition groups across in the apical and buccal sides of the crypts. The permanent dentition group had a significantly lower trabecular number across all sides when compared to the mixed dentition group (*p* < 0.001, Table 7).

Lastly, trabecular spacing differed significantly across the dentition groups in the apical and lingual sides (*p* ≤ 0.001, Table 6), but not on the buccal side (*p* = 0.171, Table 6). Trabecular spacing was significantly lower on the lingual side of deciduous group when compared to mixed dentition group (*p* < 0.001, Table 7). There were no significant differences in trabecular spacing on the apical side between the deciduous and mixed dentition groups (*p* = 0.08, Table 7). Trabecular spacing was significantly lower on the apical side of the permanent dentition group when compared to the mixed dentition group (*p* < 0.001, Table 7). No significant differences were observed on the lingual sides between mixed and permanent dentition groups (*p* = 1.00, Table 7).

**Table 6:** Kruskal–Wallis test results comparing trabecular micro-architecture parameters across dentition stages for apical, buccal, and lingual sides of dental crypts

Parameter	Crypt side	Deciduous	Mixed	Permanent	H	*p* - value
BV/TV	Apical	169.86	160.00	276.56	73.58	≤ 0.001
	Buccal	186.80	232.19	255.57	22.41	≤ 0.001
	Lingual	185.29	174.14	270.73	48.11	≤ 0.001
BS/BV	Apical	311.19	299.67	187.44	88.10	≤ 0.001
	Buccal	335.54	281.02	181.61	119.85	≤ 0.001
	Lingual	333.97	292.91	182.92	118.30	≤ 0.001
Tb.Th	Apical	162.30	174.72	276.90	74.90	≤ 0.001
	Buccal	136.96	186.88	286.10	113.35	≤ 0.001
	Lingual	131.68	177.68	292.69	133.21	≤ 0.001
Tb.N	Apical	299.80	271.11	198.08	54.05	≤ 0.001
	Buccal	321.10	303.95	183.29	107.01	≤ 0.001
	Lingual	354.12	281.25	177.62	15.58	≤ 0.001
Tb.Sp	Apical	252.80	300.23	212.65	23.29	≤ 0.001
	Buccal	225.12	210.91	243.24	3.53	0.171
	Lingual	180.90	263.56	254.89	28.14	≤ 0.001

The values presented for the crypt sides for all the dentition groups are mean ranks. Kruskal-Wallis H statistics and p-values are then presented for each trabecular parameter and side of the crypt across the three dentition groups. BV/TV, bone volume fraction; BS/BV, bone surface to bone volume ratio; Tb.Th, trabecular thickness; Tb.N, trabecular number; Tb.Sp, trabecular spacing. Statistical significance was set at *p* < 0.05.

**Table 7:** Dunn’s post-hoc pairwise comparisons of trabecular micro-architecture parameters between dentition groups across apical, buccal, and lingual crypt sides

Parameter	Group vs. group	Post-hoc sig. (0.05)		
		Apical	Buccal	Lingual
BV/TV	Deciduous vs. mixed	1.00	0.11	1.00
	Mixed vs. permanent	< 0.001	< 0.001	< 0.001
BS/BV	Deciduous vs. mixed	1.00	0.04	0.18
	Mixed vs. permanent	< 0.001	< 0.001	< 0.001
Tb.Th	Deciduous vs. mixed	1.00	0.06	0.11
	Mixed vs. permanent	< 0.001	< 0.001	< 0.001
Tb.N	Deciduous vs. mixed	0.55	1.00	0.003
	Mixed vs. permanent	< 0.001	< 0.001	< 0.001
Tb.Sp	Deciduous vs. mixed	0.08	1.00	< 0.001
	Mixed vs. permanent	< 0.001	0.30	1.00

The* p*-values presented for pairwise comparisons are post-hoc adjusted values after significant Kruskal-Wallis tests. Bonferroni correction was done to account for multiple comparisons. Statistical significance was set at *p* < 0.05

## Discussion

In the assessment of the changes in micro-architecture of the alveolar region of the postnatal and subadult human maxilla in relation to dental development and eruption, the internal architecture of the maxilla changed from a porous to a more compact appearance when comparing across the stages of tooth development and eruption. Another key finding was the increased deposition of bone along the alveolar region leading to increases in the bone volume fraction and trabecular thickness from the youngest to the oldest dentition group. The available bone surface reflected through the bone surface to bone volume and trabecular number parameters, decreased as the deciduous dentition was replaced with the permanent dentition. These findings may be important in informing educators, researchers and potentially health care practitioners of the micro-architectural changes that take place in the alveolar region of the maxillae of juvenile individuals.

In the current study when considering the deciduous dentition group, the maxillary alveolar bone was more porous in appearance as reflected by a higher bone surface to bone volume ratio as well as trabecular number. The higher trabecular number may be the reason for the higher bone surface to bone volume, i.e., there is a higher bone surface to bone volume ratio because there are more trabeculae along the buccal, lingual, and apical sides of dental crypts in the deciduous dentition group. In contrast, a reduced bone volume fraction was observed within the deciduous dentition, which showed as a lower amount of bone despite a higher bone surface available. This is further confirmed by the lower trabecular thickness observed within this dentition group. The porous nature of the maxillary alveolar bone may reflect the general growth of the maxilla because the first bone that is formed embryologically is woven and consists of many islands of soft connective tissue [47, 58]. The woven bone transitions into lamellar bone during the late stages of intrauterine life and the first years of postnatal life [47]. This process of bone formation as well as subsequent periods of bone growth appear to coincide with a transition in the functional demands emanating from the early functional environment, i.e., suckling and mastication. Thus, the transition in function may be a potential contributor to the observed general remodeling of the maxilla. As such the remodeling of the alveolar bone of the jaws may be attributed to the general skeletal development, the developing and erupting dentition and the function related forces that are transferred from the developing teeth to the alveolar bone [26, 42, 58].

During early development, the bony crypts of the alveolar region of the maxilla are wide open on the buccal side [13]. This suggests that the lingual and the apical regions are more advanced in their development when compared to the buccal side of the dental crypts. The difference in the timing and degree of development between the sides of the dental crypts may explain the higher bone volume fraction and lower trabecular number on the apical and lingual sides when compared to the buccal side observed in the deciduous dentition group in the current study. The pressure exerted by the tongue and the cheeks on the lingual and the buccal sides of the maxilla, respectively, are also significantly different [64]. In a study by Thüer et al. [64] where the pressure of the tongue and the cheek at rest and during chewing and swallowing were measured, the results showed that the pressures exerted on the lingual side were almost double when compared to the buccal side [64]. This increased pressure on the lingual side may further explain the higher bone volume fraction on the lingual side in this deciduous dentition group, although these exerted forces resulting from the tongue and cheeks were not explored in the current study [47, 69, 71].

The emergence and eruption of the deciduous dentition occurs in unison with functional demands of the masticatory system, namely suckling and mastication. During the early postnatal period (0–12 months), suckling is more prevalent and may play a partial role in maxillary alveolar bone remodeling [16]. Breastfeeding includes the functioning of the lips, which are brought close to the gingival arch, widening it anteriorly, and the tongue pressing the nipple against the palate. Bottle suckling on the other hand involves the tongue pushing upwards against the palate [8], which may also partially play a part in the remodeling of the crypt on the lingual side in the deciduous dentition group. Deciduous dentition group individuals also transition to solid foods that require mastication [10], and therefore the alveolar bone of the maxilla may experience functional demands that are likely to influence bone micro-architecture by remodeling. Another factor that may potentially contribute to changes in bone micro-architecture in the deciduous dentition group is bruxism. Bruxism is a condition where individuals grind and clench teeth and is more prevalent and extensive in young children [56]. Bruxism may start as early as one year postnatally but usually begins around four years of age [13]. This condition can result in excessive wear and attrition of teeth, damage of the periodontal tissue, and movement of teeth [13, 56], which could subsequently influence remodeling of the alveolar bone [50].

As the dentition develops and erupts, the alveolar bone of the maxilla remodels to accommodate the associated developmental changes [58]. When comparing crypt sides of the deciduous dentition group and the mixed dentition groups there were fewer statistically significant differences than when comparing the mixed versus the permanent dentition. Broadly, a change from a more porous to a less porous trabecular micro-architecture was observed. This change may be attributed to the general growth of the skeletal system, which also includes the state of dentition across the deciduous and mixed dentition groups [43]. In the mixed dentition group, a relatively lower bone surface to bone volume ratio was observed on the buccal side of the maxilla, which was inversely proportionate to trabecular thickness, while a lower trabecular number was observed on the lingual side. This may be indicative of more alveolar bone deposition when compared to the deciduous dentition, which was presented as increased bone volume fraction when overall crypt was considered. Additionally, there was a significant increase in trabecular spacing on the lingual side of the maxilla of individuals with mixed dentition. The mixed dentition group is characterized by the shedding of deciduous dentition and their replacement by permanent dentition [57, 58]. Thus, there is a need for space as individuals grow and the dentition increases in size and number. The maxillary alveolar bone also accommodates this increase in size and number of teeth by remodeling and elongation of the maxilla in a mesial direction [18, 58]. The incisive fissure also plays a role in accommodating the increased volume of dentition in the maxilla [29, 58]. It is during this period of the shedding of dentition where the most dynamic changes in the shape and size of the maxillary alveolar bone occur [44]. Unlike the deciduous dentition group, the individuals in the mixed dentition group may also experience more complex mastication and functional demands due to nutritional needs that are associated to general growth [52].

When comparing the mixed dentition and the permanent dentition groups, the skeletal age which also includes state of dentition may be the contributing factor to the micro-architectural differences between the two dentition groups. In the mixed dentition, the deciduous dentition is shed and the roots as well as some trabecular bone are resorbed [47, 58, 69]. This bone resorption during tooth shedding may play a role in the observed micro-architectural differences between the mixed and the permanent dentition groups. The thickening of the trabeculae as individuals grow up and dentition develops and erupts, observed as higher bone volume ratio as well as lower bone surface to bone volume in the permanent dentition group when compared to the mixed dentition group, may also be caused by the general bone remodeling with a positive balance, i.e., there is more bone added than resorbed [11, 52].

In the permanent dentition group, the bone is more matured, and this is presented as high bone volume fraction and thicker trabeculae. The nutritional and functional demands associated with this dentition group may be contributors to the observed compact micro-architecture of the alveolar bone [7, 23, 43, 68]. The fully matured state of the alveolar bone in the permanent dentition group is maintained by the tooth–periodontium–bone complex, also referred to as tooth–bone crosstalk [44, 48]. The components of this complex function to disperse functional loads that are exerted on the maxilla and maintain the homeostasis of the alveolar bone [6, 48]. The importance of this tooth-bone crosstalk and relationship between the alveolar bone and teeth is illustrated by the resorption of alveolar bone when teeth are lost [1, 58]. This resorption of the alveolar bone after the loss of teeth has been observed in older individuals [28, 54, 58, 65].

Malocclusions are prevalent in both children and adults and are regarded as a third priority in oral health, after caries and periodontal disease [2]. These malocclusions may be corrected using orthodontic treatments such as dental braces. Dental braces function to move teeth to their normal functional position in the alveolar bone [5]. The success of dental implants is influenced by several factors such as bone mineral density, bone volume, and trabecular micro-architecture [46, 66]. While dental implants are not common in children with deciduous dentition, they may be needed in severe cases where loss or absence of teeth may result in loss of function and disturbances in the normal growth of the alveolar ridge [53]. The information on the trabecular micro-architecture of the maxilla provided by this study may potentially provide a baseline for further insights on the quality of alveolar bone and the degree of bone growth at different stages of dental development.

While this study provides useful information about the changes in micro-architecture of the maxilla in relation to dental development and eruption, there were some limitations. The mixed dentition group was comparatively small due to limited sample availability. The included 9 individuals were the maximum that could be included from the skeletal collection that was used for this research. The small sample size may have reduced the statistical power to detect small differences between dentition groups. As such, differences involving the mixed dentition should be considered and interpreted with caution. Despite the small sample size, the mixed dentition group still represents a distinct and important developmental period which is characterized by active dentition transition and remodelling of the maxilla. As such, the group was not removed or combined with either the deciduous or the permanent dentition groups. The mixed dentition results could be viewed as preliminary and would benefit from validation using a larger sample size. The study sampled skeletal remains, and therefore medical records of the individuals were not available. This could lead to the potential inclusion of maxillae from individuals with systemic diseases that can affect bone density and micro-architecture such as cystic fibrosis [51]. Although sex specific changes were not assessed in the current study, it has been shown that permanent teeth erupt earlier in girls when compared to boys due the hormonal differences and early onset of maturation in girls [19]. Sex specific differences may affect craniofacial development and trabecular bone architecture. As such, the possibility that some of the observed differences reflect sex specific differences rather than developmental stage alone cannot be excluded. The available sample was sourced from a limited skeletal collection, and the number of females was relatively small, therefore sex specific analyses were not conducted. Future studies with larger and sex-balanced samples would enhance current knowledge on sex related differences concerning the bone micro-architecture.

Kim et al. [32] demonstrated that the anterior and posterior maxillary trabecular thickness is approximately 200 μm (0.2 mm). The relatively low resolution of the scans in the current investigation, as indicated by large voxel size of 125 μm (0.125 mm), has been shown to influence trabecular measurements obtained and is a limitation of the present study. Christiansen [14] demonstrated that trabecular thickness is highly sensitive to scan voxel size, additionally, it has been shown that even a small change in voxel size significantly alters trabecular thickness measurements, demonstrating differences of up to 236% depending on the scanner used [67]. Tayman et al. [63] reported that larger voxel sizes artificially overestimate bone volume fraction and trabecular thickness while underestimating trabecular number. However, trabecular number and trabecular separation were found to be relatively less sensitive [14]. This may be because the boundaries of thin structures cannot be adequately resolved and low resolution yields an overestimation of trabecular thickness because the minimum measurable thickness is limited by the voxel size [9]. In the current investigation, trabecular thickness showed lower repeatability when compared to the other trabecular parameters which may be explained by the relatively low resolution of the scans. Additionally, as all the trabecular measurements were obtained from the same segmented VOIs, the lower repeatability is unlikely to reflect variations in VOI placement only. The lower repeatability values may be related to the greater sensitivity of trabecular thickness calculations to minor variations in segmentation. As a result, although the observed patterns of trabecular thickening across dentition stages are informative, the lower repeatability nature of trabecular thickness means uncertainty of the measurement and should be considered with caution.

Cortical and trabecular bone were not segmented as separate compartments. To minimize cortical bone inclusion, VOIs were manually positioned within the trabecular compartment and away from the outside cortical bone of the alveolar process. The size of each VOI was adjusted according to each anatomical region within the alveolar process to ensure inclusion of trabecular bone only. While every effort was made to exclude cortical bone, minor inclusion of cortical bone cannot be ruled out completely. We acknowledge that this may have influenced the measurements of the trabecular parameters. The scanning parameters were set for the whole skull and not for specific dental crypts, and hence the higher resolution. Future studies may consider more focused approaches when scanning the volumes of interest in order to bring the resolution of the smallest sample closer to that of a bigger sample to achieve a similar resolution range. The cross-sectional nature of the study may yield information about the dentition group differences but not necessarily progressive changes in micro-architecture as observed with longitudinal observations with dental development and eruption. In future, a longitudinal study using a low dose radiation modality such as cone beam CT [32, 45], may be used to assess the changes and differences in micro-architecture observed over the periods of dental development and eruption.

The micro-architectural changes observed in this study included a change from a more porous alveolar bone to a more compact alveolar bone as individuals’ age, deciduous teeth are shed, and permanent teeth move into their functional positions. There is an increase in the deposition of bone as dentition advances, and this leads to the thickening of trabeculae. There are also site-specific changes in the micro-architecture of the maxilla such as anterior versus posterior regions and buccal versus lingual side. The patterns of micro-architectural changes observed in the maxilla between the dentition groups in this study reflect the general development and growth of the maxilla, state of dentition, as well as the complex functional environment of the oral cavity.

## Data Availability

The data that was collected for this study was under an ethical clearance for the use of skeletonized materials from body donors housed in the school of Biomedical Sciences, Department of Anatomical Sciences in the University of the Witwatersrand. The collected data is, which included micro-architecture parameters, and would not include donor information per ethics approval, is available on request via the corresponding author.
